# Case Report: Utilizing AI and NLP to Assist with Healthcare and Rehabilitation During the COVID-19 Pandemic

**DOI:** 10.3389/frai.2021.613637

**Published:** 2021-02-12

**Authors:** Jay Carriere, Hareem Shafi, Katelyn Brehon, Kiran Pohar Manhas, Katie Churchill, Chester Ho, Mahdi Tavakoli

**Affiliations:** ^1^ Department of Electrical and Computer Engineering, University of Alberta, Edmonton, AB, Canada; ^2^ School of Public Health, University of Alberta, Edmonton, AB, Canada; ^3^ Neurosciences, Rehabilitation, and Vision Strategic Clinical Network, Alberta Health Services, Calgary, AB, Canada; ^4^ Department of Occupational Therapy, University of Alberta, Edmonton, AB, Canada; ^5^ Cumming School of Medicine, University of Calgary, Calgary, AB, Canada; ^6^ Faculty of Medicine and Dentistry, University of Alberta, Edmonton, AB, Canada

**Keywords:** COVID-19, artificial intelligence, natural language processing, smart health, neuromusculoskeletal rehabilitation

## Abstract

The COVID-19 pandemic has profoundly affected healthcare systems and healthcare delivery worldwide. Policy makers are utilizing social distancing and isolation policies to reduce the risk of transmission and spread of COVID-19, while the research, development, and testing of antiviral treatments and vaccines are ongoing. As part of these isolation policies, in-person healthcare delivery has been reduced, or eliminated, to avoid the risk of COVID-19 infection in high-risk and vulnerable populations, particularly those with comorbidities. Clinicians, occupational therapists, and physiotherapists have traditionally relied on in-person diagnosis and treatment of acute and chronic musculoskeletal (MSK) and neurological conditions and illnesses. The assessment and rehabilitation of persons with acute and chronic conditions has, therefore, been particularly impacted during the pandemic. This article presents a perspective on how Artificial Intelligence and Machine Learning (AI/ML) technologies, such as Natural Language Processing (NLP), can be used to assist with assessment and rehabilitation for acute and chronic conditions.

## 1 Introduction

At the time this article was published, there were over 33 million confirmed COVID-19 patients globally, with 1 million deaths being reported (Johns Hopkins University, 2020) in over 188 countries and territories. The COVID-19 pandemic has had a profound effect on societies and healthcare systems worldwide. To address the pandemic, governments and healthcare providers have had to rethink how healthcare is delivered. COVID-19 spreads rapidly from direct or close human-to-human contact, and around 15–30% of infected individuals are asymptomatic with a large percentage of people having only mild symptoms (He et al., 2020; Tuli et al., 2020). Without a COVID-19 vaccine or proven antiviral treatment, public health policy has focused on social distancing to prevent and contain the spread of COVID-19. Healthcare systems have been forced to take drastic actions to mitigate the risk of infection and to ensure adequate healthcare system capacity. In-person treatment and healthcare delivery has therefore been reduced, or canceled, for high-risk and vulnerable populations, particularly those with comorbidities.

This change in healthcare policies and priorities caused the treatment of non-emergent (chronic or non-life-threatening) conditions to be deferred into the future. While this shift has allowed for focusing healthcare resources to address the immediate needs of the pandemic, healthcare systems had to delay and defer non-emergent treatments to mitigate or reduce the risk of COVID-19 infection to vulnerable populations in healthcare settings. Some of the vulnerable populations, who have been identified as a high-risk category for developing more severe and life-threatening COVID-19 infections, include the elderly, those with disabilities, or multiple comorbidities (Bartolo et al., 2020). The COVID-19 pandemic forced healthcare providers and healthcare systems worldwide to reduce or limit less-urgent healthcare services, such as rehabilitation services for people with acute and chronic diseases and disorders (Prvu Bettger et al., 2020). For some patients, this delay in treatment is inconvenient but not substantially detrimental. For other patients, a delay or pause in treatment can significantly impair recovery and reduce effectiveness.

The deferral of rehabilitation therapies is undesirable due to diminished patient physical and psychological outcomes, and increases the burden on the healthcare system in the future to address this growing backlog (Prvu Bettger et al., 2020; Tavakoli et al., 2020). During the COVID-19 pandemic, rehabilitation has gained significant importance. Rehabilitation is required to address the needs of those with acute and chronic conditions and to support recovery for individuals who have had severe COVID-19 infections requiring long-term intensive care and respiration support. Rehabilitation for post-COVID patients has been shown to be taxing on healthcare systems, with the average cost of rehabilitation services for post-COVID patients being roughly twice the cost of rehabilitation services for non-COVID conditions (Iannaccone et al., 2020).

In this time, when healthcare resources are being strained due to the pandemic, artificial intelligence (AI) and machine learning (ML) methods can be utilized to assist healthcare workers and healthcare delivery (Tavakoli et al., 2020). This article will provide a brief review and perspective on the use of AI/ML technologies and systems that can aid in the assessment and treatment of acute and chronic musculoskeletal, neurological and other conditions. These AI/ML technologies can be used to complement in-person appointments with clinicians, occupational therapists, and physiotherapists. As an example of such a system, a case-study outline of our work on an AI/ML and Natural Language Processing (NLP) system for a telephone-based Rehabilitation Advice Line will also be presented. With future waves of the COVID-19 pandemic expected, these technologies can also provide continuity of care when in-person appointments present too much of a risk. Additionally, beyond the immediate needs of the pandemic, the deployment of these systems will continue to be of benefit for providing care for remote and rural populations.

This paper is laid out as follows. Section 2 will cover an overview of AI and ML systems that have been applied to assisting with healthcare, including systems developed to address the COVID-19 pandemic. Section 3 discusses the use of AI/ML methods, particularly natural language processing (NLP), for assisting with rehabilitation assessment and treatment. Section 4 introduces our work using a combined ML-NLP system to analyze clinical data collected by a phone-based rehabilitation advice line during the pandemic. Section 5 presents a brief decision about the utility and concerns when using AI/ML systems within healthcare, with concluding remarks given in Section 6.

## 2 Artificial Intelligence for Healthcare and COVID-19

**FIGURE 1:**
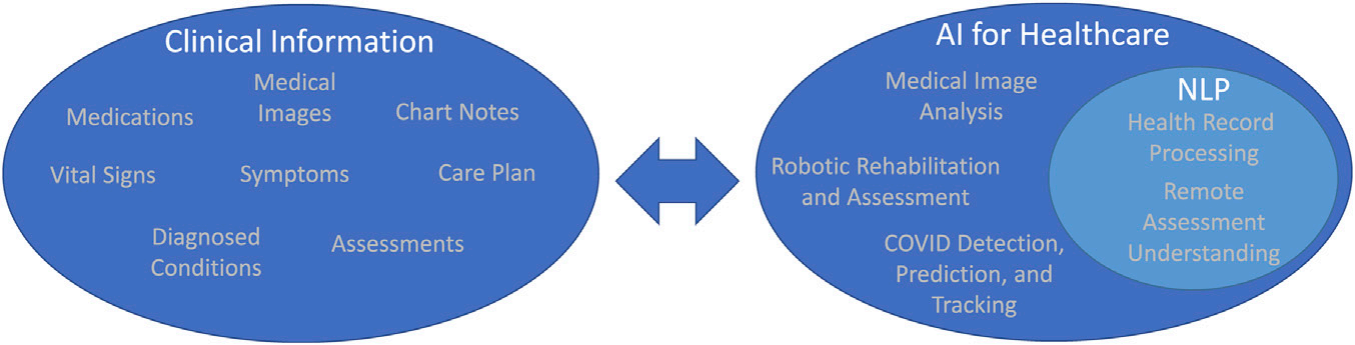
Examples of clinical information that can be processed by AI algorithms and example AI use cases within healthcare.

AI/ML techniques have been widely researched and deployed before the pandemic to aid clinicians, nurses, and healthcare workers in various healthcare tasks. Assisting with medical image based diagnosis and assessment is one such task that AI/ML technologies have been extensively researched and developed for. During the pandemic, existing and novel systems have been developed and deployed to address the particular challenges of COVID-19. These systems can provide predictions about the growth and spread of COVID-19 using AI/ML methods to assist with prevention/containment measures and can include the use of advanced robotic technologies (Tavakoli et al., 2020). Figure 1 shows the relationship between clinical data that can be processed by AI/ML systems and example use cases for AI/ML systems during the COVID-19 pandemic.

### 2.1 Medical Image Processing

AI/ML algorithms have been widely used to aid in medical image processing. Several reviews in the literature, written before the pandemic, show the widespread interest, research, and adoption of AI/ML technologies for medical image processing for a variety of imaging modalities (Shen et al., 2017; Maier et al., 2019). Deep learning and deep neural network (DNN) methods have been explored to assist with segmentation of anatomical features (or areas of interest) in x-ray, CT, MR (Lundervold and Lundervold, 2019), and other medical imaging modalities. These segmented anatomical features can create and train diagnosis and health-outcome prediction systems for a large number of patient conditions.

AI/ML technologies can enhance medical images, giving physicians and healthcare workers superhuman vision by allowing them to detect patterns or small features in medical images, which would otherwise be imperceptible (Shen et al., 2017; Maier et al., 2019). AI/ML image enhancement tools can highlight or provide clearer visualization of diagnostically relevant structures in medical images. Assisting with interpretation of medical images, particularly for diagnostic purposes, obviously benefits the healthcare system. To address the need for rapid diagnosis of COVID-19 patients and to gauge the impact and severity of a patients’ infection, these ML-based image enhancement and segmentation techniques were used to detect the presence of COVID-19 lung infections in x-ray and CT images (Panwar et al., 2020). By analyzing patterns and minute differences in a large dataset of patient images, patterns can be found. These patterns could provide an early warning system for those coronavirus cases that will become the most serious.

### 2.2 COVID-19 Modeling, Prediction, and Tracking

Knowledge of the growth and trends of a pandemic are required for prevention and containment. AI/ML methods can intelligently use official data (such as from COVID-19 task forces) or indirect data (such as from wearable fitness trackers) to predict cases in different administrative regions.

Punn et al. (2020) used COVID-19 data from the John Hopkins database to train a predictive ML model. The dataset consisted of daily case reports and daily time series summary tables. Predictions were made about total cases for the next 10 days from attributes such as province/state, country/region, last update, last known confirmed cases, recovered cases, and deaths. The prediction will allow decision making based on transmission growth, such as increasing the period or extent of lockdown, executing sanitation procedures, or providing additional healthcare resources.

Aside from direct detection of COVID-19 infections using tests, it is known that acute infections can cause a measurable change to an individual’s vital signs. For instance, resting heart rate trends in the population can indicate the presence of infection. (Radin et al., 2020) evaluated if population trends of seasonal respiratory infections, such as influenza, could be identified through wearable sensors (Fitbit) that collect resting heart rate and sleep data. Sensor data from Fitbit users in 5 US states was shown able to estimate the level of influenza-like illness rates at the state level (as reported by the CDC), using binomial and autoregressive models. The same methodology can be used to predict the spread of COVID-19 and future pandemics.

## 3 Artificial Intelligence for Rehabilitation Assessment and Treatment

There are a few modalities under which rehabilitation and assessment can be undertaken while allowing for adequate isolation and social-distancing. One of the primary advantages of these technologies is that they allow for hands-off treatment and assessment of persons with acute and chronic conditions, which is paramount with the social isolation restrictions during COVID-19.

### 3.1 Rehabilitation Robotics

One modality that has been explored in the literature is to use robotics for assisting with assessment and rehabilitation. The area of robotics for rehabilitation has seen significant development over the past three decades. Robots are able to provide the repetitive, high-intensity, interactions with patients necessary for rehabilitation (Voelker, 2005), without being subject to stress, fatigue, or injury like human beings. Robotic rehabilitation systems are highly sensorized, providing occupational and physiotherapists with high-quality objective data to assess the extent of a person’s condition, disability, or monitor rehabilitation progress. A significant amount of research has been done on robotic rehabilitation systems to make them safe and provide effective and efficient rehabilitation.

Robotic systems for rehabilitation therapy were initially explored in the late 1980s (Voelker, 2005; Van der Loos et al., 2016). Robotic rehabilitation systems have been used to assist with upper-limb and lower-limb rehabilitation and assessment. (Khalili and Zomlefer, 1988) used two double-link planar robots that were coupled with a patient’s lower limb to provide continuous passive motion for rehabilitation. In 1988, (Hogan et al., 1992) developed the MIT-MANUS, an upper-limb rehabilitation device for shoulder-and-elbow therapy. Development of upper-limb rehabilitation systems continued with devices such as the Mirror-Image Movement Enabler (MIME) robotic device, which improved muscle movements through mirror-image training (Lum et al., 2004), and the Assisted Rehabilitation and Measurement (ARM) Guide, which functions both as an assessment and rehabilitative tool (Reinkensmeyer et al., 2014). More general robotic rehabilitation systems, not limited to just upper-limb or lower-limb rehabilitation, began to emerge in the 2000s. These robotic devices allowed rehabilitation for areas such as the wrist (Williams et al., 2001), hand, and finger Worsnopp et al. (2007) for the upper-limb, and gait and ankle training (Colombo et al., 2000; Deutsch et al., 2001) for the lower limb. More recently, robots designed for training patients to perform activities of daily living (ADLs) have been developed (Guidali et al., 2011; Mehrholz et al., 2012). Newer work on robotic rehabilitation systems has focused on incorporating AI/ML technologies into these robotic systems to automatically tune the amount of assistance or resistance they provide during rehabilitation therapy. (Najafi et al., 2020; Tao et al., 2020) used AI/ML technologies to provide more effective robotic rehabilitation by learning, and replicating, the amount of assistance a physiotherapist provides for an individual patient. The work of (Fong et al., 2020) incorporated machine learning to perform functional capacity evaluation and provide rehabilitation.

### 3.2 Natural Language Processing in Healthcare

Natural language processing (NLP) is the branch of ML focused on obtaining information representations by analyzing text and speech data. NLP, or speech processing and speech understanding technologies, have become ubiquitous in consumer products, particularly cell phones and smart speakers. Recent achievements of NLP include automatic speech recognition, information extraction, and image captioning (Esteva et al., 2019). These recent achievements are being applied to develop clinical voice assistants to transcribe patient visit information into their electronic health records (EHR). This technology is designed to reduce the amount of time a clinician spends on documentation, which can increase the time and capacity of a clinician to work with patients directly during the pandemic.

Another increasingly popular use is of NLP pipelines that preprocess EHR and then find and classify disease-relevant keywords for early detection of various diseases, most notably cancer, neural and cardiac ailments (Meystre and Haug, 2006; Jiang et al., 2017). ML is used to predict and analyze the performance of alternate treatment options for stroke patients and to predict the likely outcome for each patient given their medical history. (Melton and Hripcsak, 2005) used the NLP system MedLEE to analyze discharge summaries. This analysis predicted if a patient was likely to suffer from adverse effects, and this prediction was compared to the New York Patient Occurrence Reporting and Tracking System (NYPORTS). The system processed all inpatient cases with electronic discharge summaries for two years and was shown to outperform the traditional reporting system. Similarly, another NLP search approach was used to identify postoperative surgical complications from a comprehensive EHR containing clinical notes, microbiology reports, and discharge summaries at six Veteran Health Administration centers from 1999 to 2006 (Murff et al., 2011). NLP-based methods provide an additional surveillance opportunity, but utilizing information already present in clinical notes and discharge summaries. Using the same principle of clinical assistants, IntelliDoctor, an AI-based medical assistant android app, develops a profile of the user based on symptoms and medical history to predict future medical concerns (Gandhi et al., 2019). This concept is being extended to develop a comprehensive clinical assistant that can provide initial screening before referring patients to doctors to reduce patient-doctor interactions during the pandemic (Jensen et al., 2012). NLP methods can be employed to provide recommendations for specialized healthcare to those most at risk during pandemics using the text and information in their medical records. These predictions help increase the capacity of healthcare systems and can identify populations most at risk during the pandemic. An example of such a system was demonstrated by DeCaprio et al. (2020) utilizing existing medical datasets (e.g., pneumonia, influenza, acute bronchitis, upper respiratory infections) as COVID-19 proxies.

To further improve the accuracy of these clinical assistants, work has been done to reduce biomedical text ambiguity, through the use of context, such as in (Liu et al., 2001; Schuemie et al., 2005). Information extraction systems, when applied to EHRs, can consist of a tokenizer, sentence bound detector, POS tagger, morphological analyzer, shallow parser, deep parser, gazetteer, named entity recognizer, discourse module, template extractor and template combiner (Meystre et al., 2008). Using the same principle of clinical assistants, IntelliDoctor, an AI-based medical assistant android app, develops a profile of the user based on symptoms and medical history to predict future medical concerns.

## 4 Rehabilitation Advice Line: Discussion of a Case-Study

Alberta Health Services (the healthcare authority for the province of Alberta, Canada), has launched a novel telehealth service to address the rehabilitation needs of those with acute and chronic musculoskeletal, neurological, and other conditions impacted by the pandemic. This Rehabilitation Advice Line (RAL) is a telephone service that allows patients and caregivers to speak directly with rehabilitation clinicians and professionals. The RAL is the first of its kind in Canada, was launched on May 12, 2020, and is a free service for all Albertians over the age of 18.

The RAL is staffed by occupational therapists and physiotherapists to assist and assess persons remotely, and provides improved access and continuity of care during these uncertain times. Assistance provided by the RAL includes helping patients locate appropriate services in their geographical area, provide condition specific exercises, self management advice, or education to address their rehabilitation needs. This wayfinding is particularly helpful for individuals who had their rehabilitation treatment stopped due to COVID-19, or to individuals who were unable to start rehabilitation therapy due to the pandemic. The RAL system allows the clinicians to share referrals and clinical advice with other members of the person’s healthcare team (e.g., primary care physicians). The RAL forms a part of a broader Health Link telephone service which provides free advice and health information within Alberta. The phone infrastructure and data storage for the RAL provided by Health Link.

While the RAL was implemented to address the immediate needs of patients with rehabilitation needs during the COVID-19 pandemic, the RAL aims to remain in place post-COVID. Long-term, the RAL will continue to act as a resource for patients to access immediate rehabilitation advice and guidance. Patients phoning the RAL will also be provided with referrals to available rehabilitation providers and services which are open for in-person and/or virtual visits. The RAL will continue to serve as an important resource post-COVID, particularly for the remote assistance it offers for patients in rural areas in Alberta and small urban centers with limited access to rehabilitation services.

### 4.1 Natural Language Processing Processing of RAL Clinical Notes

When a patient or caregiver phones into the RAL, clinical notes are entered into an online charting platform by the occupational and physiotherapists. These clinical notes contain key information about the patients, such as their age, location, and gender along detailing the patient’s rehabilitation concerns. We propose the use of NLP and ML technologies to assist with analyzing the information contained in these clinical notes (after anonymization). The call notes consist of unstructured data that can be classified into three categories: History including previous patient diagnoses, medications, and existing symptoms; Action taken by the RAL advisor during the call including discussion of current symptoms (including pain, weakness, or difficulty performing ADLs, etc.), subjective over-the-phone assessment, and cause of the condition (if it was caused through injury); Disposition detailing the advice provided or service referrals given to the patient. By capturing this information, the RAL provides a means of monitoring and providing assistance to individual patients.

**FIGURE 2:**
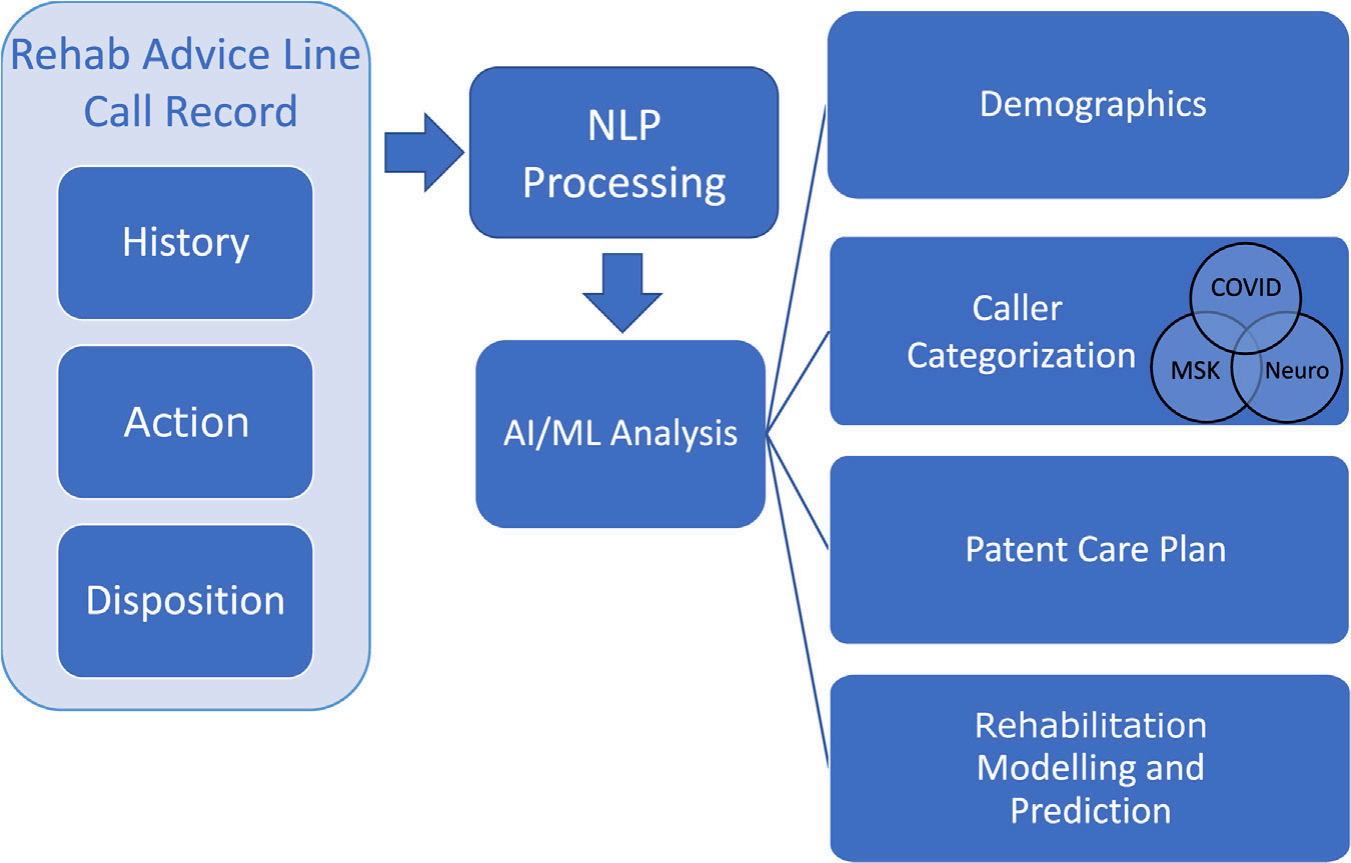
Proposed NLP-ML processing pipeline for rehab advice line call records.

An NLP-ML system has been designed as a case-study to analyze the public health impact of the RAL, user engagement with the RAL, and to provide public health monitoring and prediction of future healthcare resource needs. Along with traditional rehabilitation assessment metrics that have been collected during patient calls and surveys, our NLP-ML system will provide deeper insight into the data collected by the RAL. This insight will include: automatically capturing demographic data; categorizing the reason for the call as resulting from musculoskeletal, neurological, COVID, or other conditions; analysis of the disposition to better understand the patient care plan; and predictive modeling of areas where rehabilitation services will be needed in the future. As shown in Figure 2, the NLP-ML system consists of two main components: the NLP-based preprocessing of clinical notes and an AI/ML-based system for modeling and analyzing the collected data. Apache cTakes (Savova et al., 2010) is being used for NLP processing of the clinical notes to convert them to a machine-readable format. cTAKES is able to process and provide context from these notes, including highlighting the patient’s condition and medical history (including any injuries or medications), subjective assessment results, and the advice provided to them. Preliminary work has shown that the NLP system is capable can correctly identify salient keywords within the clinical notes (e.g., total knee replacement, multiple sclerosis, fractures, etc.). Our work on developing a ML system to distil salient public health information using a large set of these analyzed clinical notes is ongoing.

## 5 Discussion and Future Research

We have provided a number of examples that show the utility of AI/ML systems, in theory, for assisting with healthcare. In practice however, there are a number of factors which must be addressed in the future to enable the adoption of AI/ML systems outside of research environments. One set of factors that should be addressed, are the safety and accuracy when using AI/ML systems for healthcare data analysis. For some healthcare tasks, such as medical image analysis, AI/ML systems have been widely explored and have become increasingly accurate, performing nearly as well as human clinicians (Shen et al., 2017; Lundervold and Lundervold, 2019; Maier et al., 2019). The success of AI/ML in the image analysis domain can be attributed to the wide availability of high quality, comprehensive, and extensively annotated datasets. In other domains, such as NLP processing of electronic health records, there is an absence of publically available annotated datasets which can be used to develop and validate NLP systems (Kersloot et al., 2020). Due to this, there is limited information about the accuracy of NLP healthcare data analysis systems within the literature and it is difficult to compare the existing systems within the research (Kersloot et al., 2020). The development of publicly available challenge NLP healthcare datasets and better metrics for analyzing the accuracy of such systems is an area which should be worked on by researchers in the future.

In addition to the accuracy and safety of AI/ML systems, one other set of factors which should be carefully considered and discussed by researchers in the future are the ethics, privacy, and security when using AI/ML for healthcare data analysis. These factors are critical to consider when developing systems which work on identifying healthcare data, NLP systems for example. New technologies, like wearable/phone sensors, provide a wealth of new data which can be used to augment traditional clinical patient assessments, providing new insights into the day-to-day activities and symptoms of patients. The privacy and ethical use of this data needs to be discussed and addressed when developing novel healthcare AI/ML solutions. Within the COVID-19 pandemic, the balance between ethical/privacy concerns and public health assistance was a critical consideration for the various smartphone COVID-19 notification apps deployed across the world (Bradford et al., 2020).

## 6 Concluding Remarks

Healthcare systems and healthcare delivery have been significantly affected by the COVID-19 pandemic. With social distancing and isolation policies to continue until new treatment options and vaccines are widely deployed, there is a need to discuss how new and existing technologies can assist healthcare systems during this challenging time. In this perspective paper we have discussed the use of AI/ML technologies to assist with the assessment, diagnosis, and treatment of acute and chronic musculoskeletal, neurological, and other conditions during the COVID-19 pandemic. We have provided examples of AI/ML technologies applied to areas such as medical image analysis, robotic rehabilitation and assessment, and NLP systems which allow for remote, hands-off, treatment and assessment of persons with acute and chronic conditions. We have also provided an overview of our ongoing work to help the healthcare system better analyze, quantify, and understand information recorded during calls to a Rehabilitation Advice Line. As further waves of the pandemic are expected, it is important to highlight how using AL/ML technologies can be deployed to provide new public health insights using existing medical history data and new data captured during remote healthcare sessions during the pandemic.

## Data Availability

The original contributions presented in the study are included in the article, further inquiries can be directed to the corresponding author.
